# Dr. H. D. Schmidt

**Published:** 1888-12

**Authors:** 


### OBITUARY.

Dr. H. D. Schmidt.—All readers of the *Journal and Examiner* will learn with deep regret of the death of Dr. H. D. Schmidt, of New Orleans. His valuable papers have been among the very best contributions to this journal. Though a great physical sufferer and in straightened pecuniary circumstances, he has, for years carried on, unaided, investigations which rivaled the best laboratory work. In many departments of histology and pathology his name will not soon be forgotten. Some few, perhaps, will appreciate how much this cost. We have plenty and to spare of men of worldly wisdom and business acumen, but we cannot afford to lose one of his spirit. Peace to his ashes. L. C.

